# Isokinetic knee muscle strength comparison after enhanced recovery after surgery (ERAS) versus conventional setup in total knee arthroplasty (TKA): a single blinded prospective randomized study

**DOI:** 10.1186/s40634-023-00604-0

**Published:** 2023-04-15

**Authors:** Julia Goetz, Günther Maderbacher, Anna Gerg, Franziska Leiss, Silvia Dullien, Florian Zeman, Matthias Meyer, Jan Reinhard, Joachim Grifka, Felix Greimel

**Affiliations:** 1 Department of Orthopedics, University Medical Center Regensburg, Asklepios Klinikum Bad Abbach, Kaiser-Karl-V.-Allee 3, 93077 Bad Abbach, Germany; 2 Center for Clinical Studies, University Medical Center Regensburg, Franz-Josef-Strauss-Allee 11, 93053 Regensburg, Germany

**Keywords:** Enhanced recovery after surgery (ERAS), PROMS (patient reported outcome measures), Total knee arthroplasty (TKA), Knee isokinetic muscle strength, Biodex

## Abstract

**Purpose:**

Total knee arthroplasty (TKA) combined with the concept of enhanced recovery is of continued worldwide interest, as it is reported to improve early functional outcome and treatment quality without increasing complications. The aim of the study was to investigate isokinetic knee muscle strength after cemented TKA in combination with an enhanced recovery after surgery (ERAS) compared to a conventional setup.

**Methods:**

In the single blinded prospective randomized study, 52 patients underwent navigated primary cemented TKA within an ERAS (*n* = 30) or a conventional setup (*n* = 22). Preoperatively, five days and four weeks after surgery isokinetic knee muscle strength with BIODEX-type measuring device (peak torque in Nm, work in Joules and power in Watt) and subjective patient-related outcome measures (PROMs) were investigated.

**Results:**

The ERAS group showed significantly better outcomes in knee flexion at 180°/s (peak torque (Nm) *p* = 0.047, work (J) *p* = 0.040 and power (W) *p* = 0.016) 5 days postoperatively. The isokinetic measuring at knee extension 60°/s and 180°/s demonstrated no significant difference. The PROMs showed that patients were satisfied with the postoperative results in both groups. After 4 weeks, there was no longer a significant difference in isokinetic measuring at knee extension and flexion between the ERAS and conventional group.

**Conclusions:**

TKA with the concept of ERAS improves excellent isokinetic outcome and patient satisfaction. The isokinetic muscle strength measurement can help patients and surgeons to modify expectations and improve patient satisfaction.

## Introduction

Total knee arthroplasty (TKA) combined with the concept of Enhanced recovery after surgery (ERAS) or also called Fast-Track is discussed extensively in the recent literature and of worldwide interest [1, 2]. The introduction of a multimodal ERAS protocol includes alteration of medical and surgical treatment details in an interdisciplinary setup, as well as optimizing logistical and organizational aspects [3]. Kehlet et al., emphasized the anesthesia and pharmacological modifications, too [4]. Multimodal evidence-based care within the enhanced recovery reduces surgical stress and minimizes physical and psychological trauma [5].

Additionally, the economic pressures dictated a need for reduction of costs in health care systems with improved efficiency [6]. Husted et al., point out that the mean length of stay (LOS) decreased from 6.3 to 3.1 days. Nevertheless, readmission rates didn’t increase [7]. In a study of 4.500 consecutive primary hip and knee replacements, the 90-day death rate has been reduced from 0.8% to 0.2% (*p* = 0.01) in the ERAS group [8]. Enhanced recovery after TKA showed no restrictions for elderly patients with comorbidities [2]. Due to mobilization on the day of surgery, the muscle loss including morbidity can be minimized. More than that, improved pulmonary function, reduced thromboembolic and gastrointestinal complications can be achieved [9]. Already in 1997, Kahlet et al. created the first approaches of an ERAS concept [10]. Since then, the studies listed above have demonstrated the benefits of enhanced recovery after TKA: reduced LOS and postoperative complications such as embolism or thrombosis and accelerated postoperative recovery.

The focus of the ERAS concept is early mobilization. Other isokinetic studies showed, that through mobilization the isokinetic muscle strength of the knee joint after TKA extensors and flexors can be improved [11, 12]. To date, there is no published study investigating the isokinetic muscle strength of the knee joint after enhanced recovery TKA, furthermore no data regarding advantages in postoperative muscular function is available. We hypothesized that ERAS patients have significantly better muscular function with an isokinetic measurement five days after cemented TKA in combination with a concept of ERAS compared to conventional setup. Furthermore, we analyzed the patient reported outcome measures (PROMs).

## Materials and methods

In this single blinded prospective randomized study, 52 patients who underwent primary cemented TKA between May 2020 until March 2021 in a single center were included. All included patients were blindly randomized preoperatively by an independent statistician, using closed envelopes. This was the reason for the different number of cases with *n* = 30 ERAS and *n* = 22 conventional patients. Only the surgeon knew to which group the patient was assigned to. In addition, the ERAS group and the conventional setup group were separated in different wards to eliminate contact and exchange. Inclusion criteria were primary cemented navigated TKA using a DePuy P.F.C.™ (Johnson & Johnson, Raynham, MA, USA) knee system due to primary or secondary osteoarthritis. Exclusion criteria were age < 18 years or > 90 years, condition following deep vein thrombosis, therapeutic anticoagulation, immobility with preoperative walking distance < 100 m with forearm crutch or wheeled walker, tumor, a prior fracture within the surgical region, flexion < 90°, Body mass index (BMI) > 50, open pre-/surgery (except knee arthroscopy), simultaneous participation in another study or refusal by the subject. The study was approved by the local Ethics Committee (approval number 19–1470-101). The study was applied in accordance with the ethical standards of the Declaration of Helsinki 1975. No changes after trial commencement were made with regard to the course of the study.

All patients were admitted according to clinic standards, thoroughly examined and informed in detail about the TKA as well as possible complications and risks. Table 1 showed a summary of the preoperative, operative, and postoperative differences between the ERAS group and the conventional group. Multidisciplinary lecture and gait training with crutches were given only to ERAS patients, preoperative. Additionally, Etoricoxib 90 mg was applied once an hour before enhanced recovery surgery as preemptive analgesia. The ERAS group underwent surgery in spinal anesthesia (prilocaine 1% hyperbaric 4 ml = 80 mg and sufentanil 10 μg as standard) with i.v. administration of dexamethasone (8 mg). In contrast, the control group received the standardized psoas-compartment-block and proximal sciatic-nerve-block (Per block: ropivacaine 0.75% 20 ml = 150 mg and prilocaine 1% isobar 20 ml = 200 mg) with 4 mg dexamethasone for nerve block. Implantation of a P.F.C.™ (Johnson & Johnson, Raynham, MA, USA) knee system via the medial parapatellar approach was performed in both groups. In contrast to conventional TKA, the ERAS group were operated without tourniquet and received local infiltration analgesia as well as subcutaneous infiltration (200 mg ropivacaine, for deep infiltration with 0.5 mg epinephrine). Administration of tranexamic acid (1 g intravenously and 2 g topically) was performed in the ERAS group. Another difference in the ERAS group was the omission of suction drains, the application of wound adhesive after wound closure and a transparent wound dressing.

**Table 1:** Summary of preoperative, operative, and postoperative differences between the ERAS group and the conventional group

	**ERAS**	**Conventional**
**Preoperative**		
Gait training with crutches	X	-
Etoricoxib 90 mg	X	-
Anesthesia	spinal anesthesia	psoas-compartment-block and proximal sciatic-nerve-block
**Surgery**		
Medial parapatellar approach	X	X
DePuy P.F.C.™ Implant	X	X
Tourniquet	-	X
Local infiltration analgesia & Tranexamic acid application	X	-
Suction drains	-	X
**Postoperative**		
WHO pain management	X and 10 mg oxycodone for 3 days	X
Full weight bearing	X	X
First Mobilization	1–2 h after surgery	first postoperative day
Physiotherapy concept	twice daily	once daily
Motor splint	twice daily	once daily
Exercise parcour	X	-

In both groups, full weight-bearing was allowed and ROM (range of motion) was not restricted. In the ERAS group, mobilization began as soon as peripheral sensory and motor function were obtained, usually 1–2 h after surgery. After cardiovascular stimulating and thrombosis prophylaxis exercises, the first walking exercises with crutches were performed under physiotherapeutic supervision. The aim for the day of surgery was a walking distance of at least 50 m. In contrast, mobilization after conventional TKA started from the first postoperative day after the prolonged sensory and motor function restrictions have relieved. Pulling the suction drains was on the second postoperative day. Subsequently, standardized physiotherapy concept and motor splint were performed twice daily in the ERAS group and once daily in the conventional group. Physical therapy included mobilization, muscle strengthening, thrombosis and pneumonia prevention. From the first postoperative day, a parcour was used for the ERAS group patients to increase the intensity of movement. The ERAS exercise parcour consists of gait training, various muscle strengthening exercises, and instructions to improve coordination. Also, a mirror wall with a support bar on the ward was installed at the ERAS ward. Here, patients could repeat the exercises several times a day independently and under self-monitoring to reflect on their gait pattern, and to self-correct possible errors.

In our department, a standardized pain management concept was established regarding the recommendations within the World Health Organization (WHO) analgesic ladder [13] composed of the following steps: After surgery, 10 mg oxycodone was administered two times daily for three days within the ERAS group. In the recovery ward, 3 mg of piritramide was administered as needed, depending on the numerical rating scale (NRS; 0 = no pain; 10 = worst pain imaginable). Oral pain medication consists of ibuprofen (600 mg) administered three times daily and metamizole (500 mg) administered regularly four times daily. Depending on NRS values, patients can receive tramadol 100 mg (40 drops) and oxycodone 10 mg as optional additional analgesics, if needed. Importantly, the differences between the ERAS group and conventional group included only the preoperative, intraoperative and the postoperative setting during the hospital stay. After the patients were discharged, each patient was free to decide on further physiotherapeutic treatment.

### Isokinetic knee muscle strength

**Fig. 1:**
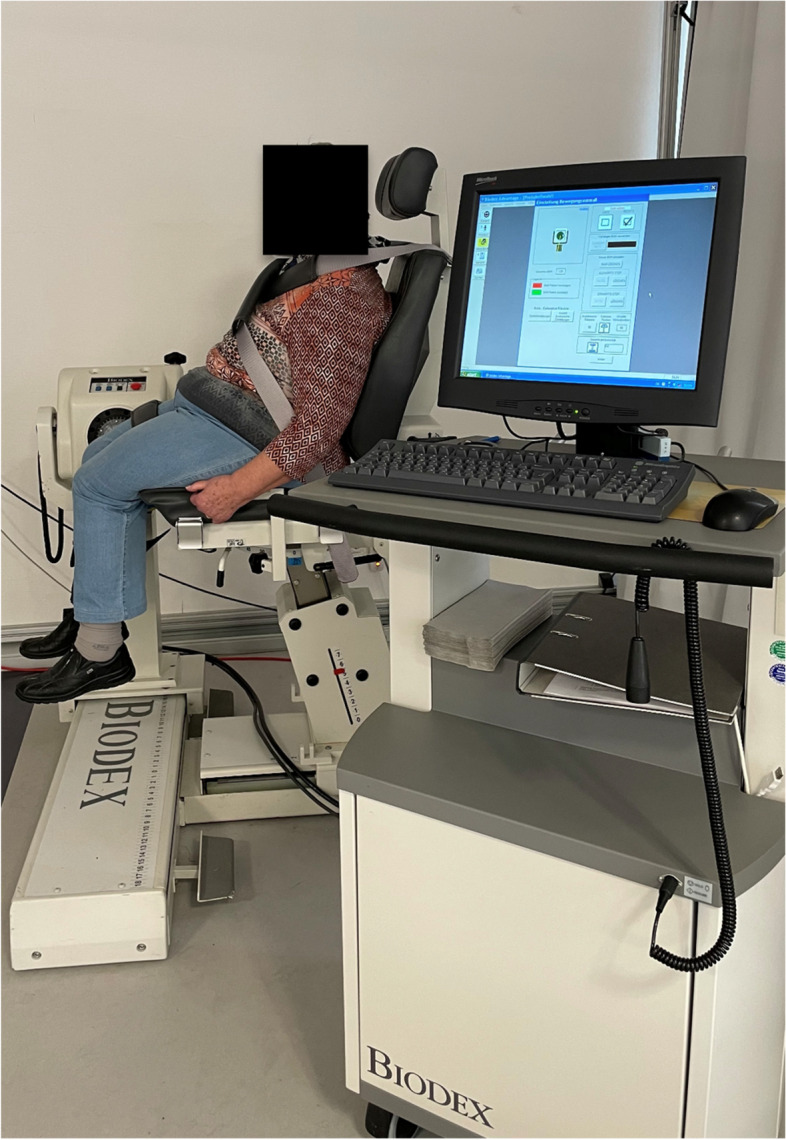
BIODEX type (Biodex System 3 Dynamometer, Biodex Medical Systems, Shirley, New York, U.S.A.).Photo from the gait laboratory of the Clinic and Polyclinic for Orthopedics at the University of Regensburg

To quantify and objectify the muscle strength around the knee, isokinetic measurements were performed using a BIODEX-type isokinetic validated measuring device (see Fig. 1): Biodex System 3 Dynamometer, Biodex Medical Systems, (Shirley, New York, U.S.A.). Drouin et al., proved the validity and reliability of the Biodex system for knee flexion and extension [14]. Baseline measurement was performed preoperatively during inpatient admission. Subsequently, patients were measured 5 days and 4 weeks postoperatively. The entire procedure of isokinetic testing followed a specified standard evidence-based protocol by the company BIODEX MEDICAL SYSTEMS to increase objectivity. Measurements were taken according to a validated test protocol pre-programmed in the Biodex device with knee flexion and extension at 60 and 180 degrees per second. In addition, a calibration of the system and a gravity correction was performed before each measurement. Due to the computer-controlled measurement and documentation, the conditions could be standardized for all subjects.

The test was started with the knee that was not operated in all cases, followed by the study leg. After informing the patient about the measurement procedure, the first step for each patient was individual positioning and fixation. First the ROM was recorded by maximum flexion and extension in the knee joint. Calibration of the dynamometer with the knee flexed to 90° followed. For the standardized warm-up and adaptation to the measurement device, each patient performed five contractions in exercise mode. On instruction of the investigator, the measurements started with knee flexion and extension at 60 degrees per second. Here, the study collective was verbally motivated to perform 10 full flexions and extensions of the leg in an alternating sequence, using maximum muscle strength throughout, starting from maximum extension. This was followed by a half-minute rest. Subsequently, the same parameters were measured again, with knee flexion and extension 180 degrees per second. The measurement of the non-operated leg was thus completed. The subsequent measurement of the study knee followed the same procedure. In addition to the peak torque in Newtonmeter (Nm), overall work in Joul (J) and power Watt (W) of the leg in flexion and extension, the Biodex System 3 Dynamometer could also calculate the peak torque relative to the body weight in Nm/kgKG and work relative to the body weight in J/kgKG.

### PROMs

To assess satisfaction, pain, quality of life and health, a subjective questionnaire was used. The following questions were analyzed before and after surgery and possible answers were: Was the operation successful in your eyes (yes / no)? Would you perform the surgery (TKA) again (yes/no)? Were your expectations of the operation met (no / light / moderate / strong / very strong)? How do you feel compared to before surgery (much better / better / same / worse / much worse)? Has your quality of life improved (no / light / moderate / strong / very strong)? How would you evaluate the function of your knee (normal / almost normal / unnormal / strongly unnormal)? Pain was assessed using the numerical rating scale (NRS) from zero to ten.

### Statistical analysis

All analyses were performed using Statistical Package for the Social Sciences (IBM SPSS Statistics for Windows, Version 27.0, IBM Corp., Armonk, NY, USA). Continuous data are presented as mean values and standard deviation (SD). Absolute and relative frequencies are used for categorical variables. The Mann–Whitney U test was performed, for comparison between the two groups. No adjustments of the significance level for multiple comparisons were performed. A *p*-value < 0.05 was considered statistically significant.

## Results

Fifty-two patients who underwent total knee arthroplasty (30 ERAS / 22 conventionally) were included. Gender, age and BMI, operated leg, ASA-score (American Society of Anesthesiologists), were assessed (see Table 2). There were no significant differences (*p* > 0.05) between the groups. The average operation time from cut to skin suture was 77.98 (± 11.21) minutes. No complications such as thromboembolic complications, fractures or revisions were recorded within the first four weeks postoperatively in either study group. Preoperatively, 100% of the patients rated the functional level of their knees as impaired or severely impaired. Five days postoperatively, there was a significant (*p* = 0.038) difference in ROM after ERAS TKA versus the conventional pathway.

**Table 2:** General, demographic data and ASA (American Society of Anesthesiologists). Categorial variables were given in absolute numbers/percent and continuous variables in mean values

General / Demographic data		ERAS	Conventional	*p*-Value
n (total)	*n* = 52	*n* = 30	*n* = 22	
Side of operation	Right	*n* = 9 (30%)	*n* = 13 (59.09%)	
	Left	*n* = 21 (70%)	*n* = 9 (40.91%)	
Gender	Female	*n* = 16 (53.33%)	*n* = 12 (52.55%)	*p* = 0.466
	Male	*n* = 14 (46.67%)	*n* = 10 (45.45%)	
Age (in years)		66.97(± 9.30)	66.95(± 8.44)	*p* = 0.498
Body Mass index (kg/m2)		31.85(± 5.55)	29.91(± 3.76)	*p* = 0.081
ASA	1	*n* = 3 (10%)	*n* = 2 (9.09%)	*p* = 0.500
	2	*n* = 24 (80%)	*n* = 18 (81.82%)	
	3	*n* = 3 (10%)	*n* = 2 (9.09%)	
	4	*n* = 0	*n* = 0	
Range of motion before surgery		94.95° (± 17.38)	92.72° (± 22.19)	*p* = 0.684
Range of motion 5 days after surgery		59.86 (± 18.41)	48.39 (19.20)	***p* = 0.038**
Range of motion 4 weeks after surgery		90,9 (± 18.54)	88,95 (± 14.57)	*p* = 0.667

**Fig. 2:**
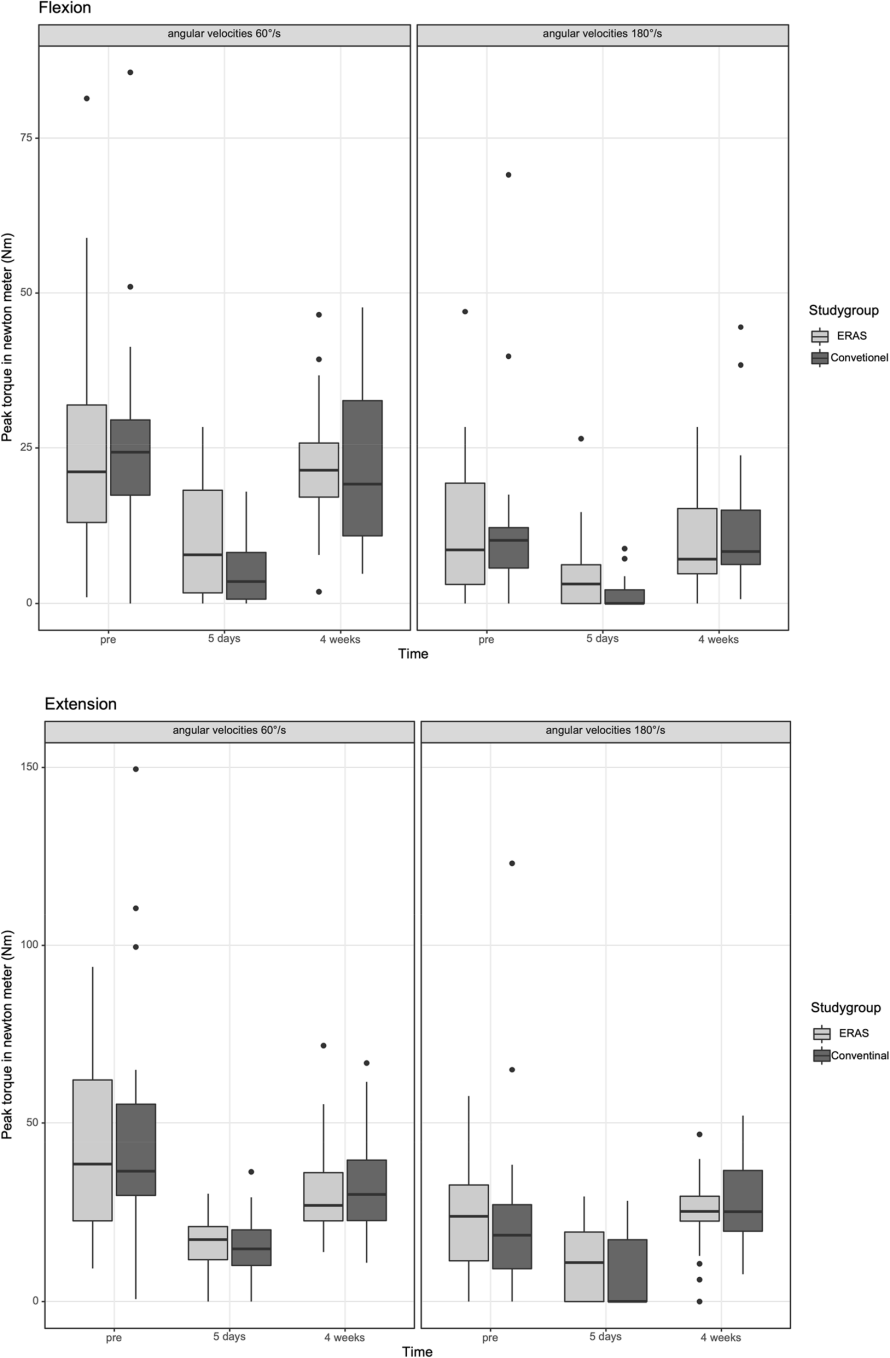
**a** and **b** The flexion (**a**) and extension (**b**) peak torque of the operated knee achieved for the two angular velocities measured (180°/s and 60°/s). The x-axis corresponds to the enhanced recovery after surgery (ERAS) to conventional setup preoperative, 5 days after surgery and 4 weeks postoperative. The y-axis corresponds to the peak t torque in newton meter (Nm)

**Fig. 3:**
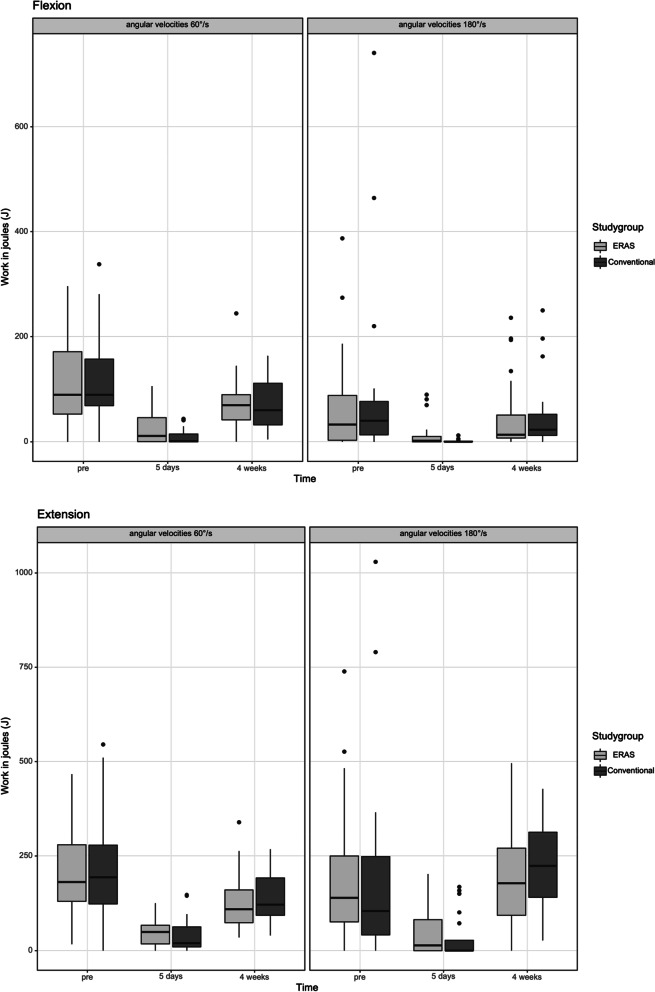
**a** and **b** The flexion (**a**) and extension (**b**) work achieved for the two angular velocities measured (180°/s and 60°/s). The x-axis corresponds to the enhanced recovery after surgery (ERAS) to conventional setup preoperative and 4 weeks postoperative. The y-axis corresponds to the work in joules (J)

**Fig. 4:**
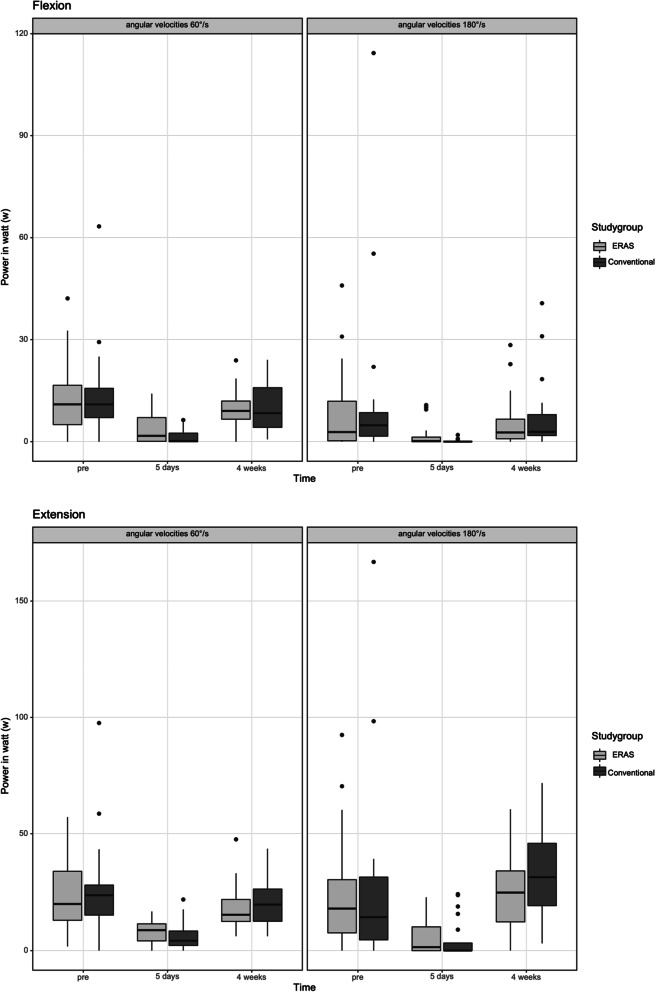
**a** and **b** The flexion (**a**) and extension (**b**) power achieved for the two angular velocities measured (180°/s and 60°/s). The x-axis corresponds to the enhanced recovery after surgery (ERAS) to conventional setup preoperative and 4 weeks postoperative. The y-axis corresponds to the power in watt (w)

The isokinetic peak torque (Nm), work (J) and power (Watt) during knee flexion and extension at 60 and 180 degrees per second were summarized in Table 3. The ERAS group performed superior outcomes during knee flexion and extension at 60 degrees per second compared to the conventional group in the subitems peak torque in Nm (Flexion: *p* = 0.074 / Extension: *p* = 0.623), work in J (Flexion: *p* = 0.072 / Extension: *p* = 0.208) and power in W (Flexion: *p* = 0.060 / Extension: *p* = 0.212) at 5 days postoperatively. Significantly better outcomes were observed in knee flexion at 180 degrees per second (peak torque *p* = 0.047, work *p* = 0.040 and power *p* = 0.016) after 5 days postoperative. Furthermore, the boxplot diagrams showed the isokinetic result of peak torque in Nm (see Fig. 2) work in J (see Fig. 3) and power in W (see Fig. 4) during knee flexion and extension at 60 and 180 degrees per second after ERAS compared to the conventional setup.

**Table 3:** Mean value and standard deviation of peak torque (Nm), work (J) and Power (W) preoperatively as well as after a follow up of 5 days and 4 weeks after surgery with enhanced recovery or conventional setup

		**ERAS**	**Conventional**	***p*-Value**
**Peak torque (Nm)**				
60°/s extension	Pre-OP	43.16 Nm (± 25.47 Nm)	47.22 Nm (± 34.71Nm)	*p* = 0.746
	5 days post-OP	16.30Nm (± 8.00 Nm)	16.08 Nm (± 8.87 Nm)	*p* = 0.623
	4 weeks post-OP	31.43 Nm (± 13.31 Nm)	33.56 Nm (± 15.43 Nm)	*p* = 0.636
180°/s extension	Pre-OP	22.99 Nm (± 15.60 Nm)	23.70 Nm (± 26.65 Nm)	*p* = 0.493
	5 days post-OP	10.57 Nm (± 10.19 Nm)	7.38 Nm (± 10.55 Nm)	*p* = 0.277
	4 weeks post-OP	24.95 Nm (± 9.99)	28.15 Nm (± 12.09 Nm)	*p* = 0.414
60°/s flexion	Pre-OP	25.93 Nm (± 18.62 Nm)	26.86 Nm (± 18.29 Nm)	*p* = 0.803
	5 days post-OP	10.61Nm (± 9.78 Nm)	5.01 Nm (± 5.47 Nm)	*p* = 0.074
	4 weeks post-OP	22.06 Nm (± 10.34)	21.24 Nm (± 12.29 Nm)	*p* = 0.484
180°/s flexion	Pre-OP	11.55 Nm (± 10.74 Nm)	12.67 Nm (± 15.06 Nm)	*p* = 1.000
	5 days post-OP	4.81 Nm (± 6.31 Nm)	1.51 Nm (± 2.51 Nm)	***p* = 0.047**
	4 weeks post-OP	10.51 Nm (± 7.92)	12.55 Nm (± 11.35 Nm)	*p* = 0.420
**Overall Work (J)**				
60°/s extension	Pre-OP	207.59 J (± 128.67 J)	215.70 J (± 146.72 J)	*p* = 0.882
	5 days post-OP	49.07 J (± 36.89 J)	40.15 J (± 45.06 J)	*p* = 0.208
	4 weeks post-OP	125.20 J (± 71.24 J)	141.82 J (± 72.42 J)	*p* = 0.355
180°/s extension	Pre-OP	187.76 J (± 177.95 J)	199.55 J (± 256.16 J)	*p* = 0.690
	5 days post-OP	49.49 J (± 64.78 J)	32.84 J (± 59.06 J)	*p* = 0.110
	4 weeks post-OP	183.17 J (± 120.48)	220.51 J (± 121.90 J)	*p* = 0.282
60°/s flexion	Pre-OP	112.92 J (± 86.61 J)	118.84 J (± 85.49 J)	*p* = 0.817
	5 days post-OP	25.86 J (± 31.26 J)	9.59 J (± 14.26 J)	*p* = 0.072
	4 weeks post-OP	69.53 J (± 50.16)	71.94 J (± 51.54 J)	*p* = 0.974
180°/s flexion	Pre-OP	64.27 J (± 89.81 J)	97.66 J (± 175.67 J)	*p* = 0.637
	5 days post-OP	12.71 J (± 24.76 J)	1.21 J (± 2.88 J)	***p* = 0.040**
	4 weeks post-OP	48.05 J (± 67.07 J)	52.48 J (± 69.30 J)	*p* = 0.471
**Power (Watt)**				
60°/s extension	Pre-OP	24.33 W (± 16.12 W)	25.46 W (± 20.64 W)	*p* = 0.897
	5 days post-OP	7.95 W (± 5.14 W)	6.52 W (± 6.38 W)	*p* = 0.212
	4 weeks post-OP	18.41 W (± 9.72 W)	21.19 W (± 11.25 W)	*p* = 0.426
180°/s extension	Pre-OP	24.45 W (± 23.63 W)	26.86 W (± 38.10 W)	*p* = 0.802
	5 days post-OP	5.73 W (± 7.75 W)	4.61 W (± 8.37 W)	*p* = 0.230
	4 weeks post-OP	24.86 W (± 15.75)	32.38 Nm (± 19.71 W)	*p* = 0.208
60°/s flexion	Pre-OP	12.82 W (± 10.39 W)	13.97 W (± 13.09 Nm)	*p* = 0.846
	5 days post-OP	3.89 W (± 4.45 W)	1.54 W (± 2.24 W)	*p* = 0.060
	4 weeks post-OP	9.64 W (± 5.65)	10.07 W (± 7.02 W)	*p* = 1.000
180°/s flexion	Pre-OP	7.80 W (± 10.70 W)	12.66 W (± 25.59 W)	*p* = 0.656
	5 days post-OP	1.67 W (± 3.14 W)	0.19 W (± 0.47 W)	***p* = 0.016**
	4 weeks post-OP	6.04 W (± 7.87)	7.68 W (± 10.77 W)	*p* = 0.568

In addition, Table 4 showed the peak torque and total work per kg of body weight at 60°/s and 180°/s in flexion and extension. Five days postoperatively, patients performed significantly (*p* = 0.030) better work per kg of body weight (J/kg) during knee flexion at 180 degrees per second.

**Table 4:** Mean value and standard deviation of peak torque (Nm) and work (J) per KG body weight preoperatively and 5 days and 4 weeks after surgery with enhanced recovery or conventional setup

		**ERAS**	**Conventional**	***p*-Value**
**Peak torque (Nm) per KG body weight**				
60°/s extension	Pre-OP	46.23 (± 24.71)	55.71 (± 41.55)	*p* = 0.585
	5 days post-OP	18.50 (± 9.87)	19.50 (± 10.47)	*p* = 0.883
	4 weeks post-OP	33.51 (± 13.34)	39.54 (± 15.81)	*p* = 0.197
180°/s extension	Pre-OP	25.29 (± 17.27)	28.87 (± 32.43)	*p* = 0.711
	5 days post-OP	11.84 (± 11.67)	8.81 (± 12.55)	*p* = 0.297
	4 weeks post-OP	27.05 (± 10.87)	33.02 (± 12.03)	*p* = 0.095
60°/s flexion	Pre-OP	27.31 (± 16.42)	31.47 (± 20.97)	*p* = 0.774
	5 days post-OP	12.30 (± 11.80)	6.04 (± 6.42)	*p* = 0.096
	4 weeks post-OP	23.70 (± 10.88)	24.82 (± 12.80)	*p* = 0.830
180°/s flexion	Pre-OP	12.69 (± 11.29)	15.57 (± 18.56)	*p* = 0.904
	5 days post-OP	5.49 (± 7.12)	1.93 (± 3.25)	*p* = 0.072
	4 weeks post-OP	12.23 (± 10.29)	14.64 (± 12.20)	*p* = 0.220
**Work (J) per KG body weight**				
60°/s extension	Pre-OP	52.83 (± 30.95)	62.27 (± 43.45)	*p* = 0.453
	5 days post-OP	14.11 (± 11.38)	12.36 (± 12.58)	*p* = 0.376
	4 weeks post-OP	31.66 (± 17.29)	38.90 (± 17.43)	*p* = 0.156
180°/s extension	Pre-OP	25.95 (± 21.93)	28.96 (± 36.30)	*p* = 0.636
	5 days post-OP	8.07 (± 9.68)	5.78 (± 9.61)	*p* = 0.237
	4 weeks post-OP	25.20 (± 15.96)	31.84 (± 14.36)	*p* = 0.135
60°/s flexion	Pre-OP	28.52 (± 18.94)	34.97 (± 24.00)	*p* = 0.453
	5 days post-OP	8.19 (± 9.67)	2.99 (± 3.97)	*p* = 0.111
	4 weeks post-OP	18.45 (± 12.28)	19.88 (± 12.67)	*p* = 0.731
180°/s flexion	Pre-OP	9.58 (± 10.99)	15.52 (± 26.32)	*p* = 0.657
	5 days post-OP	3.11 (± 5.05)	0.50 (± 1.09)	***p* = 0.030**
	4 weeks post-OP	8.07 (± 11.19)	8.69 (± 9.44)	*p* = 0.186

Both groups displayed a similar (*p* = 0.882) preoperative mean pain score of 6.00 (± 1.90, ± 1.77 respectively) points on the NRS. Four weeks postoperative the ERAS patients had a similar (*p* = 0.090) mean pain score of 2.25 (± 1.07) points compared to the conventional 3.00 (± 1.61), too. The PROMs (see Table 5) were analogically assessed four weeks after surgery. After four weeks, 100% of the patients stated that the surgery was successful in their eyes. All patients would choose a TKA again, regardless of ERAS or conventional.

**Table 5:** PROMs after a follow up of 4 weeks after surgery with enhanced recovery or conventional setup

PROM	ERAS	Conventional	*p*-Value
Was the operation successful in your eyes?	Yes = 100%	Yes = 100%	*p* = 1.000
	No = 0%	No = 0%	
Would you perform the surgery (TKA) again?	Yes = 100%	Yes = 100%	*p* = 1.000
	No = 0%	No = 0%	
Were their expectations of the operation met?	Very strong = 31.03%	Very strong = 29.41%	*p* = 0.548
	Strong = 55.17%	Strong = 47.06%	
	Moderate = 13.79%	Moderate = 11.76%	
	Light = 0%	Light = 0%	
	No = 0%	No = 11.76%	
How do you feel compared to before surgery?	Much better = 44,83%	Much better = 38.89%	*p* = 0.611
	Better = 41.38%	Better = 38.89%	
	Equal = 6.90%	Equal = 22.22%	
	Worse = 6.90%	Worse = 0%	
	Much worse = 0%	Much worse = 0%	
Has your quality of life improved?	Very strong = 13.79%	Very strong = 11.11%	*p* = 0.231
	Strong = 55.17%	Strong = 38.89%	
	Moderate = 20.69%	Moderate = 27.78%	
	Light = 3.45%	Light = 11.11%	
	No = 6.90%	No = 11.11%	
How would you evaluate the function of your knee	Normal = 14.8%	Normal = 11.11%	*p* = 0.514
	Almost normal = 70.37	Almost normal = 66.68%	
	Impaired = 14.8%	Impaired = 22.22%	
	Strongly impaired = 0	Strongly impaired = 0	

## Discussion

The most important findings of the study were that early rehabilitation with mobilization on the day of surgery (ERAS) resulted in significantly better peak torque (*p* = 0.047), power (*p* = 0.016) and work (*p* = 0.016) results at 180°/s flexion on the operated side compared to conventional group, five days postoperatively. In isokinetic tests, many studies analyzed the knee extensor and flexor strength and defined the main parameters: The peak torque (Nm) represented the maximum isokinetic knee muscle strength produced by muscle contraction of the flexors or extensors [15, 16]. The work was a measure of the muscles strength that can be performed by the traveled distance [16, 17]. The overall work was the sum of the conducted work during the defined measurement period. The power was calculated from the work performed within a specified time unit [18]. To summarize, the isokinetic measurements were suitable to ensure valid and reliable assessment of quadriceps muscle strength in TKA patients [19, 20].

This single blinded prospective randomized study – for the first time – gave significant isokinetic proof of the benefits regarding the implementation of an ERAS protocol in TKA. The importance of functional measurement was emphasized in many studies [21–23]. A variety of studies confirmed the reliability of isokinetic muscle strength measurement of the knee joint for different device types, including the Biodex System 3 Dynamometer, Biodex Medical Systems (Shirley, New York, U.S.A.), isokinetic measurement [24–26]. Our isokinetic measurement by the Biodex device showed the quick recovery with an increase in flexor and extensor muscle strength in the operated legs especially in the ERAS group. Lorentzen et al., confirmed a significant increase of the isokinetic extensor strength (14–18%) and a significant decrease of flexion strength at the operated legs [27]. Literature about isokinetic test measurement in ERAS patients compared to the conventional setup was not found. The systematic review of Husted et al., analyzed the effect of pre-operative exercise prior to and following TKA. Pre-operative exercise increased the muscle strength moderately prior to but not three months following TKA [19].

In our study, five days postoperatively isokinetic test measurement in ERAS patients were better, especially the peak torque (*p* = 0.02), the power (*p* = 0.02) and the work (*p* = 0.04) at 180°/s flexion were significantly better. However, the groups equalized after four weeks. In this regard, it must be mentioned that no different training programs were performed from rehab onwards. Hubsy et al. confirmed that intensive maximal strength training (MST) was associated with an improvement in preoperative levels of muscle strength in leg press and knee extension by 37% and 43%. Participants receiving MST of the lower extremities three times per week for eight weeks and physiotherapy session once per week showed a strength difference 12 months postoperatively [28]. Other studies with only continuous passive motion (CPM) after TKA CPM therapy didn’t show a significant positive effect on the functional outcomes [29]. Multimodal evidence-based care within the ERAS significantly enhanced postoperative recovery and reduced morbidity during the first seven days with no significant differences at 12 weeks and one, two and five years after surgery [30].

An important guideline of ERAS concept was "first better – then faster” [31]. ERAS did not only consist of early patient mobilization after surgery—the most important component considering mobilization was the perioperative educational process which resulted in a well-informed patient who will responsibly take part in postoperative physical therapy. Multimodal evidence-based care within the ERAS methodology significantly reduced medical and surgical complications and enhanced postoperative recovery [32–34]. We showed an adherence to the ERAS program was associated with no medical nor surgical complications (hip dislocation, wound problems etc.) during the inpatient stay. In a study including 6146 patients, Ripollés-Melchor et al. confirmed lower probability for moderate to severe complications in the ERAS group [35]. Also, the risk of injury to the study-patients could be reduced to a minimum due to the individual muscle strength development and the associated self-regulating adaptation of the resistance. The isokinetic movement was an apparatus-controlled, predetermined, constant movement speed against a variable resistance. The variable resistance varied individually depending on the muscle strength applied by the patient. To sum up there were several strengths to be described in this trial. We investigated not only the isokinetic muscle strength parameters including peak torque (Nm), work (J) and power (Watt) but also the clinical parameters. Patients in both study groups would perform the surgery again. We recognize that no significant differences in PROMs were demonstrated. In some recent studies, there were also no differences in PROMs or international scores (KOOS) but there were advantages for ERAS in functional outcome [22].

Another advantage of the Biodex measurement was that the patients could observe their progress. They all had the aim to achieve a good result in the measurement and were motivated by this. In addition, it was important to them to have the data explained and printed out. This confirms that patients want to be more and more informed. It was no longer enough for them just to know that they're having TKA. They want to be actively involved in their recovery [36]. An important part of the ERAS program was to involve and inform patients about the operation, the pain management, care and the rehabilitation in order to feel safe [37].

Nevertheless, patient motivation was also a limitation of the study that should not be underestimated. We used a standardized study protocol, but a lack of motivation or pain could influence the results of the measurements. All subjective parameters such as motivation, pain or physical conditions could affect the results. A limitation would also be muscular deficits or injuries thus skewing results. There were many other limitations to the study that was performed. We decided to use the BIODEX-type isokinetic measuring device. First, no other isokinetic muscle strength devices were used for comparison. Other limitations of the study were the monocentric study design and the small number of cases counting 52 study participants. Although we took care to ensure that there were no significant differences between the included patients in either study group (randomization), the distribution was not homogeneous with 30 ERAS and 22 conventional patients. Furthermore, the study could not determine which change in the ERAS concept (see Table 1) was responsible for the better outcome—but this was not the target of the study due to the widespread knowledge, that ERAS concepts are only effective when conducted consequently regarding all components. Whether individual modifications such as anesthesia, pain management, or increased mobilization by physical therapy were responsible for the effect could be questioned in further studies.

## Conclusion

It could be concluded that the isokinetic muscle strength of the knee joint flexors were significantly (*p* < 0.050) improved through the early and intensive mobilization in ERAS compared to the conventional setup, five days postoperatively. However, the ERAS program only covered the operation and the first sixth postoperative days. When the patients were discharged, they were free to decide on further physiotherapeutic follow-up. After four weeks postoperatively, no significant differences were evident. The PROMs showed that patients were satisfied with the postoperative results in both groups. Future studies could expand the ERAS program to the period after hospital discharge.

## Data Availability

The availability of data and materials is detailed described in the methods.
